# Clinical impact of serum exosomal microRNA in liver fibrosis

**DOI:** 10.1371/journal.pone.0255672

**Published:** 2021-09-10

**Authors:** Young Chang, Jae-A. Han, Suk Min Kang, Soung Won Jeong, Tom Ryu, Han Seul Park, Jeong-Ju Yoo, Sae Hwan Lee, Sang Gyune Kim, Young Seok Kim, Hong Soo Kim, So Young Jin, Seongho Ryu, Jae Young Jang

**Affiliations:** 1 Department of Internal Medicine, Institute for Digestive Research, Digestive Disease Center, Soonchunhyang University College of Medicine, Seoul, Korea; 2 Soonchunhyang Institute of Medi-bio Science (SIMS), Soonchunhyang University, Cheonan, Korea; 3 Department of Internal Medicine, Soonchunhyang University College of Medicine, Bucheon, Korea; 4 Department of Internal Medicine, Soonchunhyang University College of Medicine, Cheonan, Korea; 5 Department of Pathology, Soonchunhyang University College of Medicine, Seoul, Korea; Institut de Pharmacologie Moleculaire et Cellulaire, FRANCE

## Abstract

**Background/aim:**

We investigated alterations in the expression of serum exosomal miRNAs with the progression of liver fibrosis and evaluated their clinical applicability as biomarkers.

**Methods:**

This study prospectively enrolled 71 patients who underwent liver biopsy at an academic hospital in Korea. Exosomes were extracted from serum samples, followed by next-generation sequencing (NGS) of miRNAs and targeted real-time quantitative polymerase chain reaction. A model was derived to discriminate advanced fibrosis based on miRNA levels and the performance of this model was evaluated. Validation of the effect of miRNA on liver fibrosis *in vitro* was followed.

**Results:**

NGS data revealed that exosomal miR-660-5p, miR-125a-5p, and miR-122 expression were changed significantly with the progression of liver fibrosis, of which miR-122 exhibited high read counts enough to be used as a biomarker. The level of exosomal miR-122 decreased as the pathologic fibrosis grade progressed and patients with biopsy-proven advanced fibrosis had significantly lower levels of exosomal miR-122 (*P* < 0.001) than those without advanced fibrosis. Exosomal miR-122 exhibited a fair performance in discriminating advanced fibrosis especially in combination with fibrosis-4 score and transient elastography. In a subgroup of patients with a non-viral etiology of liver disease, the performance of exosomal miR-122 as a biomarker was greatly improved. Inhibition of miR-122 expression increased the proliferation of the human hepatic stellate cell line, LX-2, and upregulated the expression of various fibrosis related proteins.

**Conclusion:**

Exosomal miR-122 may serve as a useful non-invasive biomarker for liver fibrosis, especially in patients with non-viral etiologies of chronic liver disease.

## Introduction

Exosomes are 30–100 nm sized small membrane-enclosed vesicles that originate from internal multivesicular bodies. These are released into the extracellular space and found in various biological fluids [1]. Exosomes contain cell-type specific information, including proteins, mRNAs, and microRNAs (miRNAs), and mediate intercellular communication by shuttling these molecules to other cells. Studies have unveiled the role of exosomes in liver diseases [2]. Liver cells release and accept exosomes, which mediate intercellular communication and bring physiological and pathological changes [3]. Various studies have reported the use of exosomes as biomarkers and therapeutics for a variety of liver diseases [4–6].

miRNAs are small, non-coding RNA molecules that regulate gene expression by binding to target gene mRNAs to facilitate mRNA degradation and hinder the translation process [7]. Dysregulation of miRNA has been associated with various diseases because miRNAs play important roles in the proliferation, apoptosis, and differentiation of various cell types [8, 9]. miRNAs can exist in the bloodstream, incorporated into extracellular vesicles such as exosomes or attached to RNA-binding proteins [10]. Among the various types of circulating miRNAs, exosomal miRNAs are particularly protected from endogenous RNases that allow them to mediate intercellular communication regardless of the distance [11]. Most reports on circulating miRNAs have focused on total circulating miRNAs, and only a few studies have isolated exosomes for miRNA analyses [12]. The potential of exosomal miRNAs as biomarkers of alcoholic hepatitis, drug-induced liver injury, and hepatocellular carcinoma is well documented [4, 13–15]. However, the role of exosomal miRNAs in liver fibrosis has not been studied.

In principle, the diagnosis of liver fibrosis is established pathologically through liver biopsy, an invasive procedure associated with several complications, including abdominal pain, bleeding, and rarely, death [16]. Many non-invasive methods have been developed as alternatives to liver biopsy for evaluating liver fibrosis [17]. Transient elastography (TE) and two-dimensional shear wave elastography are representative non-invasive methods [15, 18] and magnetic resonance electrography has been recently applied to replace liver biopsy in many clinical trials [19]. Attempts have been directed to develop serological biomarkers such as aspartate aminotransferase (AST)-to-platelet ratio index (APRI) and fibrosis-4 (FIB-4) using simple biochemical blood tests [20, 21]. However, none of these non-invasive methods has replaced liver biopsy mainly owing to the associated inaccuracy. In general, these noninvasive methods cannot stage fibrosis and have much poorer positive predictive values than negative predictive values; in other words, these methods are optimized for excluding, not diagnosing, liver fibrosis [22].

In the present study, we investigated the relationship between exosomal miRNAs and liver fibrosis and evaluated their clinical potential as novel non-invasive biomarkers.

## Methods

### Study population

This study prospectively recruited consecutive patients who underwent percutaneous liver biopsy due to acute or chronic liver diseases between March 2018 and January 2019 in an academic hospital (Soonchunhyang University Seoul Hospital) in Korea. The exclusion criteria were as follows: patients under 20 years of age, those with contraindications to liver biopsies such as ascites and bleeding tendencies, patients who refused to participate in the study, or those who did not have critical data such as TE and biochemical data.

A total of 71 patients were studied, and their blood samples were obtained. Before the treatment of acute or chronic liver disease, an ultrasonography-guided percutaneous liver biopsy was performed and blood samples were collected in the same day under fasting status for more than 6 hours. Of these, exosomal miRNA sequencing was conducted in subjects who had chronic liver disease, agreed with the gene analysis, and whose blood samples were suitable for next-generation sequencing in both quantity and quality of the samples. TE was performed in the study population to measure liver stiffness by a non-invasive method other than liver biopsy. Before the procedure, patients fasted for at least 2 hours. In principle, it was performed on the same day or one day before or after the blood collection date. But in case of acute hepatitis, it was performed after acute hepatitis improved, resulting in AST and ALT levels of less than 200 IU/mL and total bilirubin of less than 3 mg/dL. Data, including individual medical status, various laboratory findings, liver stiffness measured by TE, and pathologic reports of the liver tissue, were collected.

### Exosome isolation

Exosomes from 1 mL of serum samples were isolated using the ExoQuick Exosome Precipitation Solution (System Biosciences, Palo Alto, CA, USA) following the manufacturer’s protocol. In brief, the serum was centrifuged at 3000 ×*g* for 15 min to remove cells and debris. Then, 1/4 volume of ExoQuick solution was added to the serum and the mixture was incubated at 4°C. The samples were centrifuged twice at 1500 ×*g* for 30 min, and the obtained pellet was resuspended in 100 μL of phosphate-buffered saline (PBS). Total RNA, including miRNA, was extracted from exosomes using the miRNeasy Mini Kit (Qiagen, Hilden, Germany), according to the manufacturers’ protocol. RNA was eluted in 20 μL RNase-free water.

### Next-generation sequencing (NGS) of exosomal miRNAs from human serum samples

Patient samples were processed, and 10 ng of exosomal RNA was used as an input for each library. Small RNA libraries were constructed using the SMARTer smRNA-Seq Kit (Illumina, Takara Bio, Shiga, Japan) according to the manufacturer’s guidelines. Sequencing libraries were generated by polyadenylation, complementary DNA (cDNA) synthesis, and polymerase chain reaction (PCR) amplification.

The libraries were gel-purified and validated by assessing size, purity, and concentration using the Agilent 2100 Bioanalyzer (Agilent Technologies, Santa Clara, California, USA). The libraries were quantified by qPCR according to the qPCR Quantification Protocol Guide (KAPA Library Quantification Kits for Illumina Sequencing Platforms). The quality of libraries was assessed using the D1000 ScreenTape System (Agilent Technologies, Waldbronn, Germany). Equimolar amounts of libraries were pooled and sequenced on an Illumina HiSeq 2500 instrument (Illumina, San Diego, CA, USA) to generate 101 base reads. Image decomposition and quality value calculations were performed using the modules of the Illumina pipeline. All procedures for NGS analysis were performed by Macrogen (Seoul, Korea). Clustered reads were aligned to the reference genome by miRBase 21 to identify miRNAs. The full miRNA NGS data is available at https://www.ncbi.nlm.nih.gov/geo/query/acc.cgi?acc=GSE179961.

### Analysis of differential miRNA expression

The reads were normalized by relative logarithmic expression using DESeq2 (Genome Biology Unit, European Molecular Biology Laboratory, Heidelberg, Germany). For preprocessing, miRNAs undetected from more than 50% of all samples were excluded, leaving only mature miRNAs for analysis. For each miRNA, baseMean and log-fold change values were calculated and compared between the case and control groups. A statistical hypothesis test was conducted for the comparison of these groups using the negative binomial Wald test in DESeq2. Differentially expressed miRNAs between the two groups were determined by assessing miRNAs with a |fold change| ≥ 2 and false discovery rate–adjusted P value < 0.05. We also performed hierarchical clustering analysis using complete linkage and Euclidean distance as measures of similarity to display the expression patterns of differentially expressed miRNAs that satisfy the criteria mentioned above.

### Isolation and quantification of exosomal miRNAs from human serum samples

Exosome and total RNA, including miRNA, were extracted using Total RNA Extraction Kit (INtRON Biotechnology, Seongnam-si, Gyeonggi-do, South Korea). cDNA synthesis and targeted real-time quantitative PCR (qPCR) for miRNA-122 were conducted using Maxima SYBR Green/Rox qPCR master mix 2× (Thermo Fisher Scientific, Waltham, MA, USA) and miRNA-specific primers.

### Cell lines and cell culture

An immortalized human hepatic stellate cell line. LX-2 (kindly provided by Prof. Sae Hwan Lee, Soonchunhyang University College of Medicine, Cheonan, Korea) was cultured in Dulbecco’s modified Eagle’s medium (DMEM) supplemented with 10% fetal bovine serum (FBS), 10,000 U/mL penicillin, 10 mg/mL streptomycin, and 25 μg/mL amphotericin. Cells were incubated under standard normoxic conditions (20% O_2_ and 5% CO_2_ at 37°C).

### Transfection of LX-2 cells with miR-122 inhibitor

LX-2 cells were seeded in six-well plates (8.5 × 10^4^ cells/mL) and allowed to reach 60–80% confluency. Cells were transfected with an miRNA-122 inhibitor (MI0000442; Thermo Fisher Scientific, Waltham, MA, USA) at a concentration of 150 pmoL using Lipofectamine RNAiMAX (Thermo Fisher Scientific, Waltham, MA, USA) following the manufacturer’s instructions. Lipofectamine RNAiMAX reagent was mixed at 1:1 ratio with diluted miRNA-122 inhibitor or negative control and incubated at room temperature for 5 min to generate Lipofectamine-miRNA inhibitor complexes. The complex was then added to LX-2 cells, and cell proliferation and target gene and protein expression were analyzed after 24 h.

### Cell proliferation analysis

Cell proliferation was measured based on the conversion of the colorimetric 3,4-(5-dimethylthiazol-2-yl)-2,5-diphenyltetrazolium bromide (MTT) reagent into soluble formazan by dehydrogenase enzyme found in metabolically proliferating cells using the Cell Titer 96 Aqueous One Solution cell proliferation assay (G358C; Promega, Madison, WI, USA). Following each treatment, 400 μL of dye solution was added into cells in each well of a six-well plate and incubated for 2 h. The absorbance was recorded at a wavelength of 490 nm using a Varioskan LUX Multimode Microplate Reader (Thermo Fisher Scientific, Waltham, MA, USA).

### Real-time RT-qPCR analysis

Total RNA was extracted using Total RNA Extraction Kit, and cDNA was synthesized using High-Capacity cDNA Reverse Transcription Kit (Applied Biosystems, Waltham, MA, USA). After reverse transcription, the cDNA template was amplified by PCR using SYBR green (Thermo Fisher Scientific, Waltham, MA, USA). Collagen-1A (*COL-1A*), alpha-smooth muscle actin (*α-SMA*), fibronectin (*FN1*), and transforming growth factor-β (*TGF-β*) gene expression was quantitated by real-time RT-qPCR (StepOnePlus; Thermo Fisher Scientific, Waltham, MA, USA) using Maxima SYBR Green/Rox qPCR master mix (Thermo Fisher Scientific, Waltham, MA, USA). Glyceraldehyde-3-phosphate dehydrogenase (*GAPDH*) was used as a loading control. The primer sequences are shown in S1 Table in S1 File. The expression level of each targeted mRNA was calculated as the relative intensity of the PCR product as compared to that of *GAPDH* gene using the 2^−ΔΔCt^ method. All PCR experiments were performed in triplicates.

### Immunoblot analysis

Cells were lysed on ice for 20 min using a lysis buffer and centrifuged at 14,000 ×*g* for 10 min at 4°C. For sodium dodecyl sulfate-polyacrylamide gel electrophoresis (SDS-PAGE), samples were transferred onto nitrocellulose membranes and the membranes were blotted with appropriate primary antibodies at a dilution of 1:1000. The membranes were then treated with peroxidase-conjugated secondary antibodies (Santa Cruz Biotechnology Inc., Santa Cruz, CA, USA). Bound antibodies were detected with Super Signal West Pico PLUS Chemiluminescent Substrate (ECL; Amersham, Arlington Heights, IL, USA) and developed with Kodak X-OMAT films (Kodak, New Haven, CT, USA). Primary antibodies used in this study were COL-1A (ab34710; Abcam, Cambridge, UK), α-SMA (ab5694; Abcam, Cambridge, UK), fibronectin (sc-69681; Santa Cruz Biotechnology Inc., Santa Cruz, CA, USA), TGF-β (sc-130348; Santa Cruz Biotechnology Inc., Santa Cruz, CA, USA), and GAPDH (ATGEN, Seongnam-si, Gyeonggi-do, South Korea) antibodies.

### Statistical analysis

Baseline characteristics were compared between groups using an independent sample *t*-test for continuous variables and chi-square test for categorical variables. In NGS analyses, the mean values and standard deviation (SD) of reads count and reads per million (RPM) were calculated and compared according to fibrosis grades. Outliers having values exceeding mean plus SD were considered to be excluded. The independent risk factors for advanced fibrosis were identified using univariate and multivariate Cox proportional hazards models. A model to discriminate advanced fibrosis using miRNA levels was derived by multivariate logistic regression. The performance of the variables and the model to predict advanced hepatic fibrosis was evaluated by receiver operating characteristic (ROC) curve and area under the curve (AUC) analysis and compared using the DeLong’s test. All *in vitro* experimental results were obtained from at least three independent experiments. Experimental data were analyzed by a Student’s *t*-test for comparison between groups. For all tests, differences with P values less than 0.05 were regarded as statistically significant. Statistical analyses were performed using PASW version 23.0 (IBM Corp., Armonk, NY, USA) and R version 3.5.3 (R Foundation for Statistical Computing, Vienna, Austria).

### Ethical consideration

This study was conducted in accordance with the recent ethical guidelines of the World Medical Association Declaration of Helsinki and approved by the Institutional Review Board (IRB) of Soonchunhyang University Seoul Hospital (IRB No. 2017-11-015). All subjects provided written informed consent for inclusion in the study. Medical records of the study population were fully anonymized and de-identified before analysis.

## Results

### Baseline characteristics of the study population

We collected samples from 71 patients, of which 48 (67.6%) had non-viral and 23 (32.4%) had viral etiology of liver disease. Table 1 shows the baseline characteristics of the study population. The median age of the study population was 57, and the majority (66.2%) was female. Although the liver function of the study population was preserved with median Child-Pugh score of 5, serum level of aspartate transaminase and alanine transaminase were generally elevated with median level of 75 and 70, respectively. Patients with non-alcoholic fatty liver disease (NAFLD) accounted for most of the non-viral etiology population (30 out of 48, 62.5%), autoimmune liver disease 16.7%, and alcoholic liver disease 8.3%. Remaining five patients had drug induced liver injury and two were unknown. No significant differences were observed in baseline characteristics by etiology, including baseline liver function and hepatic fibrosis represented by various scoring systems, except for platelet count, which was higher in the non-viral etiology population with a marginal statistical significance (*P* = 0.047).

**Table 1:** The baseline characteristics of the study population.

	Non-viral (n = 48)		Viral (n = 23)		Total (N = 71)		P value
Etiology					HBV: 15		
					HCV: 8		
	NAFLD: 30				NAFLD: 30		
	Alcohol: 4		HBV: 15		Alcohol: 4		
	PBC: 4		HCV: 8		PBC: 4		
	AIH: 4				AIH: 4		
	Etc: 7						
					Etc: 7		
Age	58 (44.75, 63.25)		56 (37, 65.5)		57 (40.5, 64)		0.888
Male	14 (29.2%)		10 (43.4%)		24 (33.8%)		0.24
Platelet	194 (167.5, 234.75)		169 (133.5, 200)		181 (159, 230)		0.047*
Albumin	4.28±0.45		4.10±0.47		4.22±0.46		0.142
Total bilirubin	0.65 (0.5, 1)		0.6 (0.5, 1.1)		0.6 (0.5, 1)		0.814
AST	90.5 (48.75, 131)		66 (41, 109.5)		75 (42.5, 127)		0.316
ALT	70.5 (33, 141.25)		57 (32, 146)		70 (33, 146)		0.878
PT (INR)	1.09 (1.04, 1.15)		1.12 (1.08, 1.21)		1.1 (1.04, 1.16)		0.105
Child-Pugh score	5 (5, 5)		5 (5, 5)		5 (5, 5)		0.797
MELD score	7.55 (6.87, 8.21)		7.7 (6.87, 8.21)		7.7 (7.03, 8.52)		0.353
APRI	1.02 (0.53, 2.12)		0.96 (0.54, 1.7)		1.01 (0.52, 1.92)		0.831
FIB-4	2.64 (1.53, 4.76)		2.86 (1.29, 4.97)		2.64 (1.45, 4.79)		0.937
Transient elastography (kPa)	10.3 (6.3, 14.3)		11.3 (7.85, 13.7)		10.8 (6.38, 14.3)		0.622
Pathologic grade of fibrosis	Gr 0	6 (12.5%)	Gr 0	2 (8.7%)	Gr 0	8 (11.3%)	0.15
	Gr 1	11 (22.9%)	Gr 1	2 (8.7%)	Gr 1	13 (18.3%)	
	Gr 2	14 (29.2%)	Gr 2	8 (34.8%)	Gr 2	22 (31.0%)	
	Gr 3	13 (27.1%)	Gr 3	7 (30.4%)	Gr 3	20 (28.2%)	
	Gr 4	4 (8.3%)	Gr 4	4 (7.4%)	Gr 4	8 (11.3%)	

NAFLD, non-alcoholic fatty liver disease; AIH, autoimmune hepatitis; PBC, primary biliary cholangitis; AST, aspartate aminotransferase; ALT, alanine aminotransferase; PT, prothrombin time; INR, international normalized ratio; MELD, model for end stage liver disease; APRI, AST to platelet ratio index; FIB-4, fibrosis-4

Data are expressed as mean ± standard deviation or median (interquartile range) according to normality test.

Among the study population, eight patients manifested as acute hepatitis, and sub-group clinical data were expressed in S2 Table in S1 File. Of these, three had non-alcoholic fatty liver disease, three had drug-induced liver injury, one had autoimmune hepatitis, and one remained unknown etiology. One patient who diagnosed as autoimmune hepatitis was treated with steroid after diagnostic work-up including liver biopsy and blood sample collection, and other patients received conservative management without a specific treatment.

### NGS to evaluate exosomal miRNA expression changes through liver fibrosis stages

**Fig 1:**
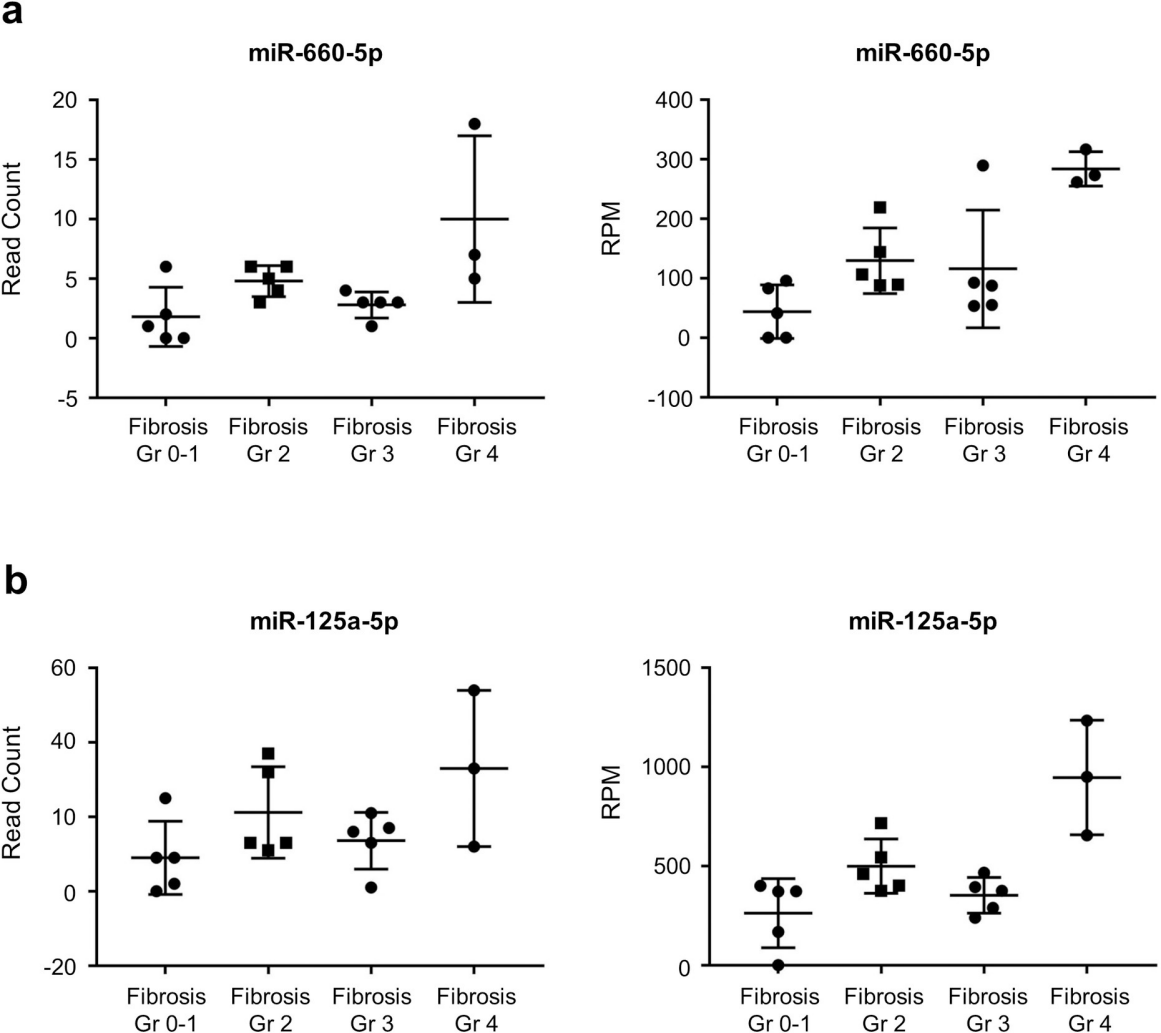
Both miR-660-5p and miR-125a-5p are differentially expressed in each fibrosis stage. Read counts and reads per million counts (RPM) by NGS according to liver fibrosis stages of (a) miR-660-5p and (b) miR-125a-5p.

To evaluate miRNAs with altered expression with the progression of liver fibrosis, we randomly selected 18 patients with NAFLD etiology. The baseline characteristics of these selected patients are shown in Table 2. NGS results (GSE179961) revealed various exosomal miRNAs that were differentially expressed according to liver fibrosis stages. We filtered exosomal miRNAs that showed at least two-fold changes and were significantly expressed (*P* < 0.05) through liver fibrosis stages (S3 Table in S1 File). Considering both average expression level and read count of each miRNA, miR-660-5p, miR-125a-5p, and miR-122 had significantly different expression according to liver fibrosis grade. The fold changes of these miRNA expression level according to liver fibrosis grade are presented in S4 Table in S1 File. The expression of miR-660-5p and miR-125a-5p showed an increasing trend with the progression of liver fibrosis (Fig 1). However, the read counts of miR-660-5p and miR-125a-5p were quite low; less than 10 and 50, respectively.

**Table 2:** The baseline characteristics of the selected patients for next-generation sequencing of exosomal microRNAs.

	Fibrosis Gr 0–1 (n = 5)	Fibrosis Gr 2 (n = 5)	Fibrosis Gr 3 (n = 5)	Fibrosis Gr 4 (n = 3)	Total (N = 18)
Age	47 ± 14.75	51.8 ± 15.96	63.6 ± 7.06	67.33 ± 7.02	56.33 ± 14.04
Female	4 (80%)	5 (100%)	5 (100%)	2 (66.7%)	16 (88.89%)
Platelet	222.5 (180, 233.5)	186 (181, 196)	180 (176.5, 268.5)	159 (138, 179.5)	191 (173.25, 227.25)
Albumin	4.3 (4.2, 4.4)	4.5 (4.5, 4.5)	4.3 (4.3, 4.4)	4.5 (4.4, 4.55)	4.4 (4.3, 4.5)
Total bilirubin	0.46 ± 0.21	0.48 ± 0.13	0.6 ± 0.07	0.97 ± 0.72	0.59 ± 0.33
AST	72.6 ± 41.38	106 ± 47.93	84.4 ± 36.84	78 ± 38.57	86.06 ± 40.25
ALT	29 (23, 33)	77 (35, 117)	70 (26, 89)	30 (27.5, 45)	34 (26.75, 86)
PT (INR)	1.05 ± 0.05	1.1 ± 0.06	1.1 ± 0.04	1.21 ± 0.05	1.1 ± 0.07
Child-Pugh score	5 (5, 5)	5 (5, 5)	5 (5, 5)	5 (5, 5)	5 (5, 5)
MELD score	7.05 ± 0.46	7.46 ± 0.67	7.49 ± 0.37	9.27 ± 1.6	7.66 ± 1.04
Transient elastography (kPa)	11.86 ± 3.98	11.02 ± 2.15	16.8 ± 3.08	20.3 ± 7.45	14.41 ± 5.17

AST, aspartate aminotransferase; ALT, alanine aminotransferase; PT, prothrombin time; INR, international normalized ratio; MELD, model for end stage liver disease

Data are expressed as mean ± standard deviation or median (interquartile range) according to normality test.

**Fig 2:**
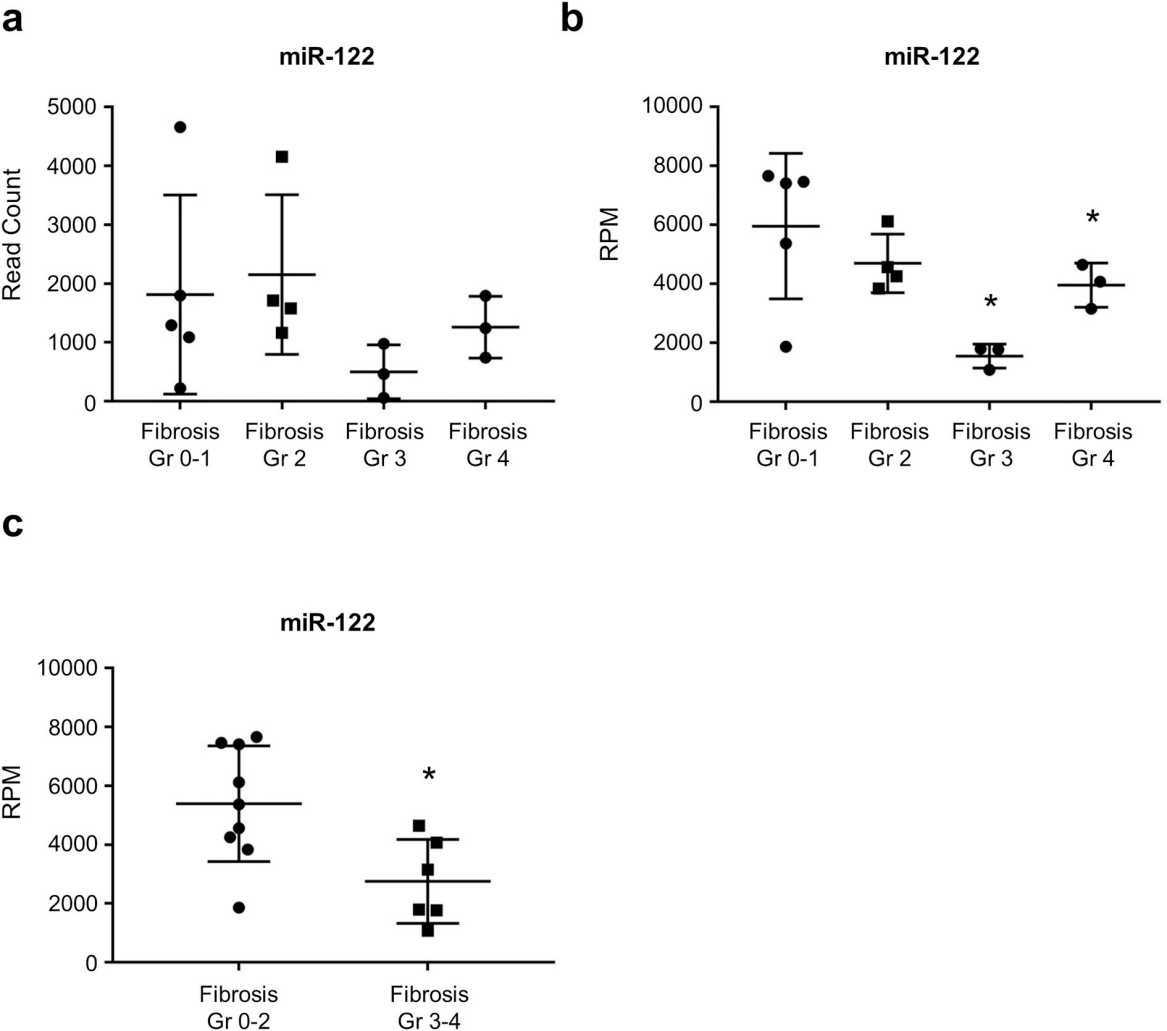
The expression of miR-122 is decreased as liver fibrosis progressed. (a) Read counts and (b) RPM counts by NGS according to liver fibrosis stages. (c) RPM of miR-122 patients divided into fibrosis stage 0–2 and fibrosis stage 3–4. Data were analyzed by omitting outliers. *: p < 0.05.

We tracked the expression of miR-122 at various stages of liver fibrosis, and observed a downward expression trend with considerably higher read counts. Analysis of the data by omitting the outliers (one in fibrosis grade 2 and two in fibrosis grade 3) revealed statistically significant differences in miR-122 expression levels at each fibrosis grade (*P* = 0.04, Fig 2 and S1 Fig in S1 File). Patients with advanced fibrosis (fibrosis grade 3–4) showed significantly lower expression levels of miR-122 than those with lower grade fibrosis (*P* = 0.02, Fig 2 and S1 Fig in S1 File).

### Exosomal miR-122 expression in liver fibrosis

**Fig 3:**
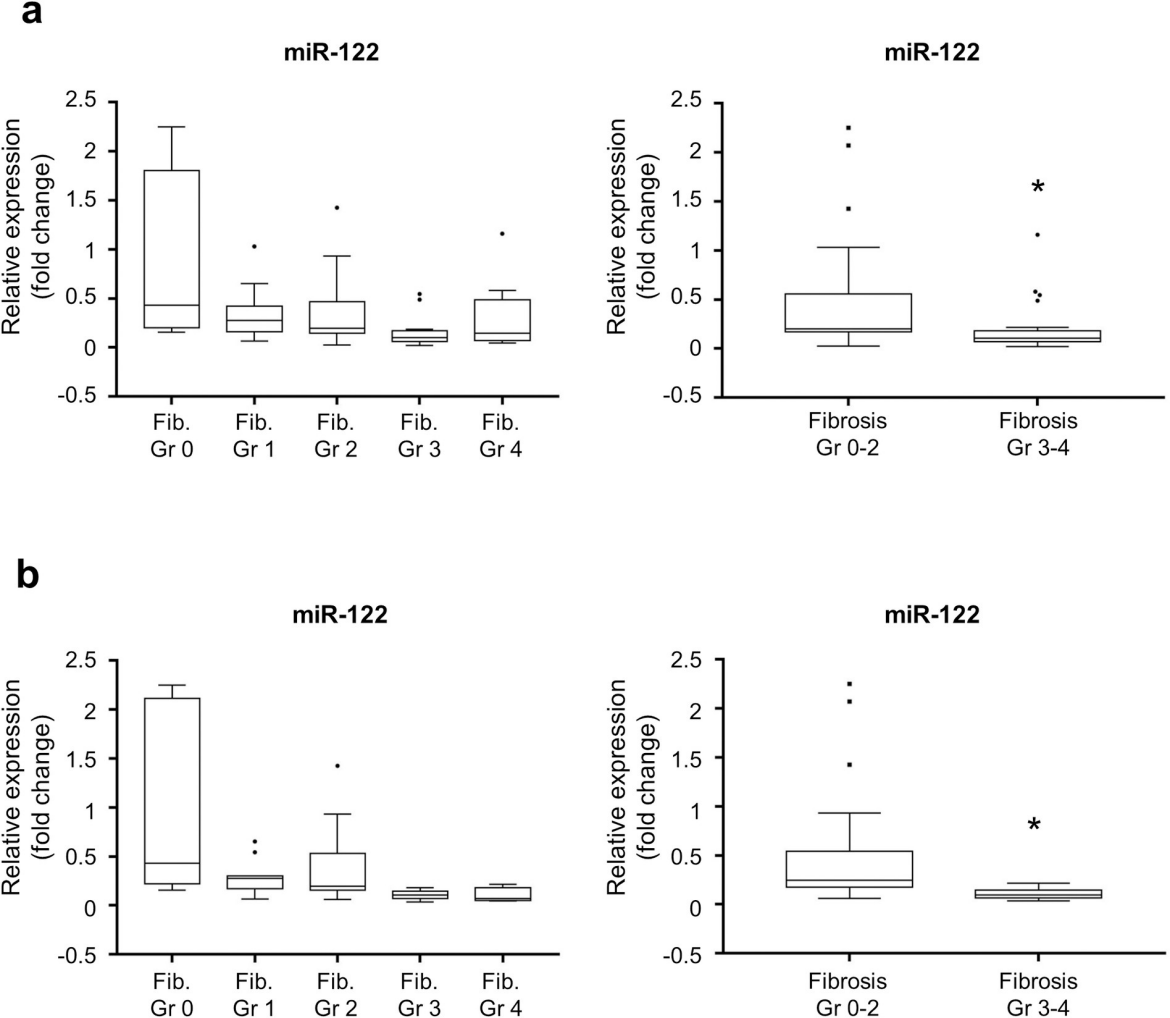
The expression of miR-122 is downregulated in advanced liver fibrosis stages. miR-122 expression was quantified in (a) total population and (b) non-viral etiological population. *: p < 0.05.

To confirm the results of NGS analysis, targeted real-time RT-qPCR for exosomal miR-122 was performed for all study population. As expected, miR-122 expression showed a decreasing trend with the progression of liver fibrosis. Patients with advanced fibrosis showed a significant decrease in the expression level of miR-122 (*P* < 0.01, Fig 3 and S2 Fig in S1 File). The decrease in miR-122 expression was more evident when only the non-viral etiology population was selected (*P* < 0.01, Fig 3 and S2 Fig in S1 File).

### Exosomal miR-122 discriminates advanced liver fibrosis

**Fig 4:**
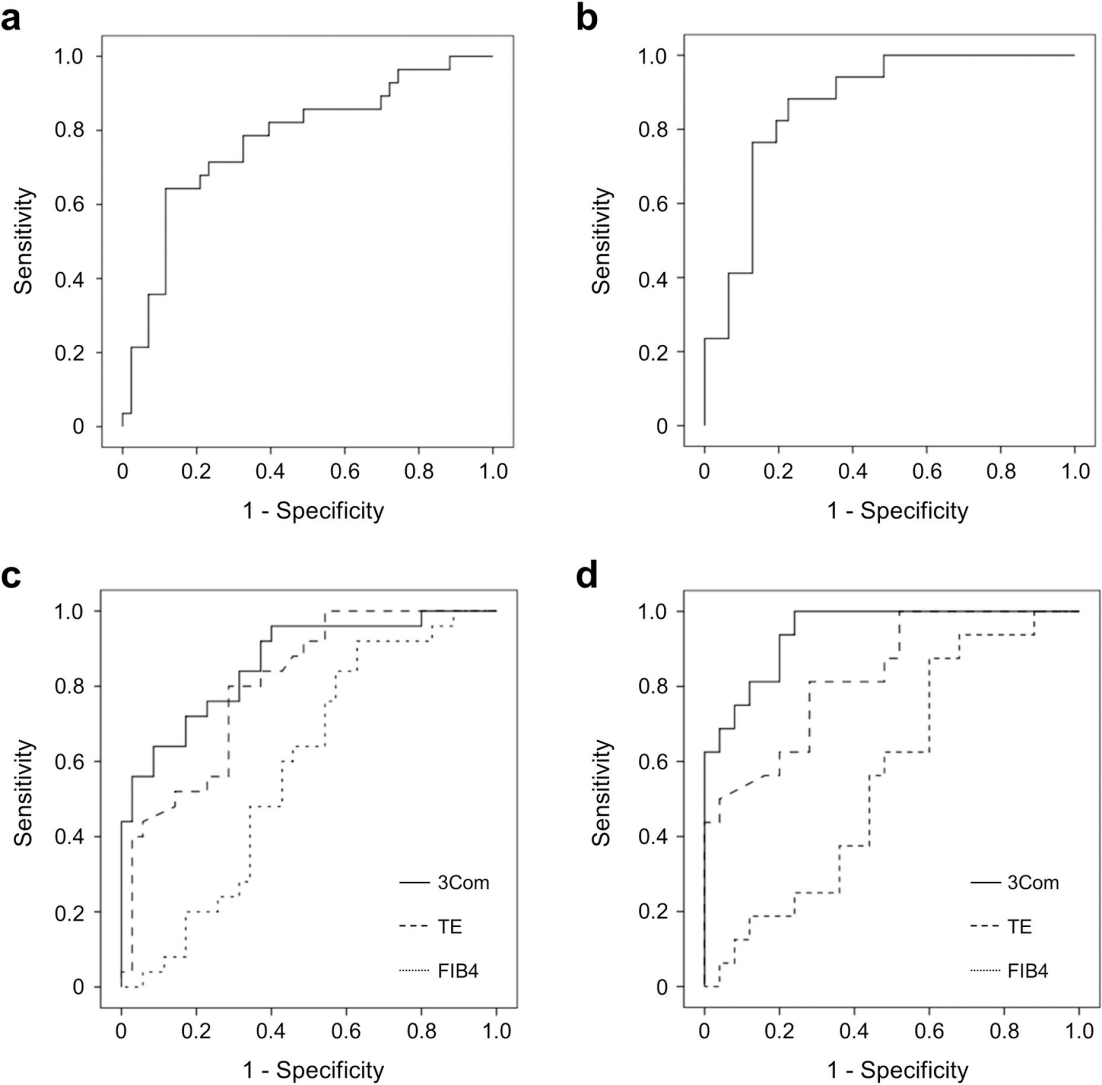
Exosomal miR-122 shows a fair discriminative ability for advanced liver fibrosis. Graphs of miR-122 ROC for (a) total population and (b) non-viral etiological population. Comparison of the ROC between combinational model using miR-122, FIB-4, and TE in (c) total population and (d) non-viral etiological population.

Exosomal miR-122 showed a fair discriminative ability in predicting advanced liver fibrosis with an AUC value of 0.77 (95% confidence interval [CI]: 0.66–0.89), which further improved to 0.87 (95% CI: 0.78–0.97; Fig 4) when limited to the non-viral etiology population. A model comprising miR-122, FIB-4 score, and stiffness measured by TE was derived using a binary logistic regression analysis (Table 3). The discriminative power for advanced liver fibrosis was higher in miR-122 model (AUC = 0.86, 95% CI: 0.78–0.95) than with FIB-4 score (AUC = 0.58, 95% CI: 0.44–0.69; *P* < 0.01) or stiffness measured by TE (AUC = 0.80, 95% CI: 0.69–0.89; *P* = 0.15) alone (Fig 4). In the non-viral etiology population, the discriminative power of miR-122 model further improved to an AUC of 0.95 (95% CI: 0.90–0.99); this value was significantly higher than that observed with FIB-4 score (AUC = 0.57, 95% CI: 0.41–0.95; *P* < 0.01) or TE alone (AUC = 0.83, 95% CI: 0.69–0.93; *P* = 0.03 Fig 4).

**Table 3:** Multivariate analysis of variables predicting advanced liver fibrosis.

Variables	Overall			Non-viral etiology		
	B	OR (95% CI)	*P*	B	OR (95% CI)	*P*
miR-122	-3.85	0.02 (0.00–1.98)	0.10	-16.81	<0.01 (0.00–0.02)	0.01
FIB-4	-0.22	0.80 (0.65–0.996)	0.046	-0.009	0.99 (0.67–1.47)	0.97
Stiffness	0.29	1.34 (1.13–1.59)	<0.01	0.29	1.34 (1.07–1.69)	0.01

OR, odds ratio; FIB-4, fibrosis-4

**Fig 5:**
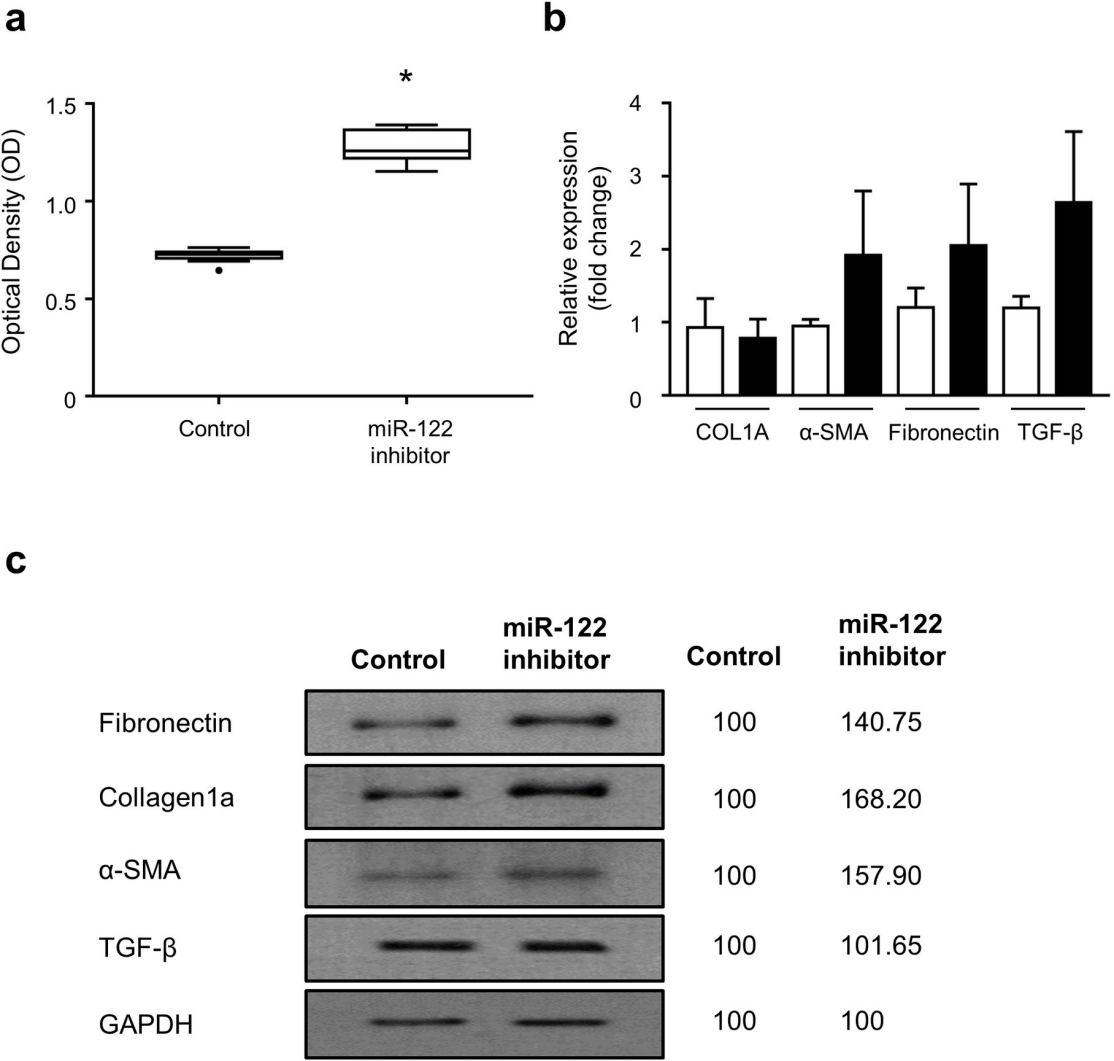
Exosomal miR-122 inhibition increases hepatic stellate cell proliferation and fibrosis marker expression. (a) MTT assay of negative control and miRNA-122 inhibitor-transfected LX-2 cells measured by optical density. (*: p < 0.05) (b) RT-qPCR analysis of *COL-1A*, *α-SMA*, *FN1*, and *TGF-β* genes in negative control and miRNA-122 inhibitor-transfected LX-2 cells. (c) Immunoblots of the corresponding proteins in (b). The numbers next to blots show their optical density ratio (%).

**Validation of the effect of miR-122 on liver fibrosis in vitro.** In the MTT assay, miR-122 expression inhibition significantly increased the proliferation of stellate cells as compared to the negative control (Fig 5). The inhibition of miR-122 expression also resulted in an increase in the mRNA expression of *α-SMA*, *FN1*, and *TGF-β*, but no significant difference in *COL-1A* mRNA expression was observed. Western blot analysis showed that the expression of COL-1A, α-SMA, fibronectin, and TGF-β increased following miR-122 inhibitor treatment (Fig 5).

## Discussion

In this study, we employed NGS to demonstrate that exosomal miRNAs, miR-660-5p, miR-125a-5p, and miR-122 were associated with the progression of liver fibrosis. We show that exosomal miR-122 has the potential to serve as a biomarker for advanced liver fibrosis. In combination with other non-invasive markers of fibrosis, exosomal miR-122 exhibits excellent discriminative power for pathologically proven advanced liver fibrosis, especially in the population with non-viral etiology. *In vitro* studies using hepatic stellate cells (HSCs) revealed the role and mechanism of miR-122 in liver fibrosis. Downregulation of miR-122 expression could promote the proliferation of hepatic stellated cells and increase the expression of various fibrosis markers.

Various miRNAs are known to modulate liver fibrosis through different mechanisms both *in vivo* and *in vitro* [23–27]. However, only a few studies have used human blood and liver tissue samples to analyze exosomal miRNAs and their effects on liver fibrosis. In this study, we prospectively collected blood samples from patients who underwent liver biopsy based on clinical needs. Although NGS revealed the increasing trend in miR-660-5p and miR-125a-5p expression with fibrosis progression, their read counts were extremely low for use as diagnostic markers. The expression of miR-122, on the contrary, showed a decreasing trend as fibrosis progressed with considerably higher read counts. The correlation between exosomal miR-122 and liver fibrosis was validated in a larger population by qPCR, realizing its potential as a novel biomarker for advanced liver fibrosis. The role of miR-122 and the underlying mechanism of action were studied with *in vitro* experiments, wherein downregulation of miR-122 expression was thought to be one of the possible causes of advanced liver fibrosis as well as a phenomenon caused by liver fibrosis progression.

While naked RNAs are rapidly degraded in the blood, exosomes protect the cargo, thereby facilitating detection and providing data consistency [28]. The levels of serum exosomal miRNAs are higher than those of serum non-exosomal miRNAs [29]. Packaging of miRNAs into exosomes is a highly selective and specific process, and numerous reports have shown that exosomal miRNAs affect biological processes in recipient cells [30]. In this regard, our study is based on miRNAs, especially those packaged in exosomes may suggest accurate and reliable clinical relevance in real practice.

miR-122 is one of the most abundant miRNAs in the human liver [31], and plays a crucial role in liver development, differentiation, and functions [32–34]. In line with the essential role of miR-122 in liver homeostasis, any alteration in the expression of miR-122 has been reported to be associated with various liver diseases such as chronic hepatitis B or C, NAFLD, and drug-induced liver disease [35–38]. Several animal experiments have shown that genetic deletion of miR-122 may lead to the progression of steatohepatitis and fibrosis, and that miR-122 expression was attenuated in a carbon tetrachloride-induced mouse model of liver fibrosis [39–41]. However, another clinical study reported that elevated miR-122 serum levels correlate with hepatic cell death and necro-inflammatory activity in chronic hepatitis C patients but not with fibrosis stage or liver function [42]. Theses discrepancies night be due to differences in etiology of liver disease. In this study, we performed NGS to discover exosomal miRNA associated with liver fibrosis in NAFLD patients, confirming that miR-122 is inversely associated with liver fibrosis grade, and validating the performance of miR-122 as a biomarker of liver fibrosis. Exosomal miR-122 has demonstrated clinical utility as a non-invasive biomarker for liver fibrosis, especially in patients with non-viral etiologies that primarily includes NAFLD. Moreover, serum miR-122 expression was recently reported as a useful marker of liver fibrosis in chronic viral hepatitis patients including chronic hepatitis C [43], and chronic hepatitis B patients [44]. In addition, it has been also reported that the expression level of miR-122 in liver tissue varies inversely with liver fibrosis progression across various liver disease etiologies [45].

The mechanism underlying miR-122–mediated regulation of liver fibrosis is still elusive. In an *in vitro* experiment using HSCs, miR-122 prevented hepatic inflammation by inhibiting lipopolysaccharide-induced cytokine production and the recruitment of immune cells to the liver [46]. Chronic hepatic inflammation results in hepatic fibrosis and cirrhosis, and HSCs play key roles in both hepatic inflammation and fibrosis [47, 48]. Accordingly, miR-122 has a potential to attenuate hepatic fibrosis by regulating hepatic inflammation mediated by various pro-inflammatory cytokines in HSCs. In addition, a recent study suggested that miR-122 controls liver fibrosis by targeting prolyl 4-hydroxolase, which is involved in collagen maturation [39]. Proline hydroxylation plays a crucial role in the stabilization of the triple helix of collagen molecule, and attenuation of prolyl 4-hydroxylase activity may lead to unstable collagen production [49]. Overexpression of miR-122 has been proposed to attenuate prolyl 4-hydroxylase expression, thereby leading to the inhibition of mature collagen-1A production. Consistent with the previous study, our results showed that miR-122 inhibition promoted liver fibrosis by increasing mature collagen-1A level. The marked increase in collagen-1A at the protein level, but not the mRNA level, following miR-122 inhibition suggests that miR-122 regulates collagen maturation instead of production. In addition, miR-122 inhibition resulted in the transactivation of HSCs and modulation of mRNA expression levels of various extracellular matrix genes, suggesting that miR-122 is involved in liver fibrosis via various mechanisms.

This study has several limitations. First, we did not include normal subjects as controls. Although there were patients with grade 0 fibrosis, they had acute or chronic liver diseases requiring a liver biopsy and were grouped together and analyzed to be equivalent to grade 1 fibrosis because their number was too small. Second, we intuitively confirmed the association between miR-122 and hepatic fibrosis using the LX-2 cell line, because HSCs play a major role in the regulation of hepatic fibrosis. However, since hepataic fibrogenesis is not an action of only stellate cells, but an interaction between various cells existing in the liver, it is recommended to utilze primary hepatocytes along with non-parenchymal cells such as Kupffer, stellate and biliary cells to reproduce hepatic fibrogenesis. Third, miRNAs are abundantly expressed in primary hepatocytes more than in hepeatic stellate cells. However, they are also expressed in HSCs [46], and moreover, exosomes play a role in trasporting miRNAs between hepatic cells, which makes it possible that hepatocytes-derived miRNAs affect the function of HSCs [50]. Therefore, down-regulation of miR-122 in hepatic cells can affect the function of hepatis stellate cells in both autocrine and paracrine manners. Further research is needed regarding changes in miR-122 levels in hepatic stellate cells during hepatic fibrosis progression. Fourth, the study population was heterogeneous since this study included both acute and chronic liver disease patients, but detailed clinical data related to treatment history were unavailable, resulting in underlying variables controled limitedly. However, the effects of drugs to the expression of miRNAs were minimized by maintaining fasting status before collecting liver tissue and blood samples. Lastly, external validation was not performed in this study. Vogt et al. have reported that miR-122 demonstrated high variability in serum from healthy volunteers, which may make miR-122 challenging for use as a prospective biomarker of liver damage or injury [51]. This study is expected to be less affected by the inter-individual variability in serum miR-122 levels, as miR-122 packed in serum exosomes was targeted rather than miR-122, which freely circulates in serum. However, since natural variability between individuals clearly exists, further validation studies, including a large number of patients, may be warranted.

In conclusion, exosomal miR-122 levels significantly decreased in pathologically proven advanced liver fibrosis. Exosomal miR-122 can serve as a clinically useful non-invasive biomarker for liver fibrosis especially in patients with non-viral etiologies primarily involving non-alcoholic fatty liver disease.

## Supporting information

S1 File (DOCX)

S2 File (ZIP)
