# Supplementary material for: Clinical impact of serum exosomal microRNA in liver fibrosis
Source: PLoS One. 2021 Sep 10;16(9):e0255672. doi: 10.1371/journal.pone.0255672 (PMC8432846; doi:10.1371/journal.pone.0255672)
Supplement: S1 File — (DOCX) [file pone.0255672.s001.docx]

**Supplementary materials**

**Table of Contents**

1. **Supplementary Table………………………………………………….…………..…….2**
2. **Supplementary Figure legend…………………………………………………………...7**
3. **Supplementary Figure…………………………………………………………………...8**
4. **STROBE Statement……………………………………………………………………...10**

**S1 Table. List of primer sequences used for qRT-PCR analysis in this study**

| Genes | Primer sequences | |
| --- | --- | --- |
| α-SMA | Forward | 5’-CCGACCGAATGCAGAAGGA-3’ |
|  | Reverse | 5’-ACAGAGTATTTGCGCTCCGAA-3’ |
| COL1a | Forward | 5’-TGACCTCAAGATGTGCCACT-3’ |
|  | Reverse | 5’-ACCAGTCTCCATGTTGCAGA-3’ |
| TGF-β | Forward | 5’-CCCTGGACACCAACTATTGC-3’ |
|  | Reverse | 5’-TGCGGAAGTCAATGTACAGC-3’ |
| Fibronectin | Forward | 5’-CAAGCCAGATGTCAGAAGC-3’ |
|  | Reverse | 5’-GGATGGTGCATCAATGGCA-3’ |
| GAPDH | Forward | 5’-AGCCACATCGCTCAGACAC-3’ |
|  | Reverse | 5’-GCCCAATACGACCAAATCC-3’ |
| miR-122 | Forward | 5’-UGGAGUGUGACAAUGGUGUUUG-3’ |

SMA, smooth muscle actin; COL, collagen; TGF, transforming growth factor, GAPDH, Glyceraldehyde 3-phosphate dehydrogenase

**S2 Table. Sub-group clinical data from the study population according liver disease etiology**

|  | Acute hepatitis (n=8) | | Autoimmune liver disease (n=8) | Metabolic liver disease (n=34) |
| --- | --- | --- | --- | --- |
| Etiology | NAFLD: 3, Drug-induced liver injury: 3  AIH: 1, Unknown: 1 | | AIH: 4  PBC: 4 | NAFLD: 30  Alcohol: 4 |
| Age | 51 ± 17.04 | | 54.38 ± 12.99 | 58 (47, 64) |
| Male | 14 (29.2%) | | 1 (12.5%) | 9 (26.5%) |
| Platelet | 173.62 ± 42.13 | | 220.25 ± 69.1 | 192 (169, 231) |
| Albumin | 3.91 ± 0.51 | | 4.09 ± 0.45 | 4.4 (4.2, 4.6) |
| Total bilirubin | 5.42 ± 5.04 | | 0.7 (0.5, 3.28) | 0.6 (0.5, 0.9) |
| AST | 515.5 (256.25, 78.25) | | 114.5 (53, 283) | 75 (44, 120) |
| ALT | 642.5 (397.5, 204.75) | | 91 (49.75, 229.25) | 55 (31, 89) |
| PT (INR) | 1.2 ± 0.2 | | 1.12 ± 0.17 | 1.1 (1.04, 1.15) |
| APRI | 9.69 ± 8.37 | | 2.58 ± 2.76 | 1.03 (0.55, 1.67) |
| FIB-4 | 6.43 ± 4.34 | | 2.56 (2.07, 3.5) | 2.64 (1.92, 4.72) |
| Transient elastography (kPa) | 7.73 ± 3.29 | | 7.37 ± 4.11 | 11.85 (7.13, 15.45) |
| Pathologic grade of fibrosis | Gr 0  Gr 1  Gr 2  Gr 3  Gr 4 | 3 (37.5%)  3 (37.5%)  2 (25%)  0  0 | 1 (12.5%)  5 (62.5%)  2 (25%)  0  0 | 5 (14.7%)  9 (26.5%)  6 (17.6%)  9 (26.5%)  4 (11.8%) |

NAFLD, non-alcoholic fatty liver disease; AIH, autoimmune hepatitis; PBC, primary biliary cholangitis; AST, aspartate aminotransferase; ALT, alanine aminotransferase; PT, prothrombin time; INR, international normalized ratio; APRI, AST to platelet ratio index; FIB-4, fibrosis-4

Data are expressed as mean ± standard deviation or median (interquartile range) according to normality test.

**S3 Table. Various exosomal microRNAs differentially expressed according to fibrosis stage**

|  |  | Fold change | *P* value |
| --- | --- | --- | --- |
| Fibrosis 2/1 | hsa-miR-27a-3p | 7.770 | 0.025 |
|  | hsa-miR-629-5p | 3.074 | 0.023 |
|  | **hsa-miR-486-5p** | **2.571** | **0.014** |
|  | **hsa-miR-16-5p** | **2.556** | **0.036** |
|  | hsa-miR-320b | 2.083 | 0.007 |
|  | hsa-miR-328-3p | -2.066 | 0.043 |
|  | hsa-miR-1273e | -2.712 | 0.040 |
|  | **hsa-miR-1273f** | **-2.827** | **0.006** |
|  | hsa-miR-409-3p | -6.621 | 0.005 |
|  | **hsa-miR-4433b-3p** | **-8.681** | **0.004** |
|  | **hsa-miR-1273a** | **-9.553** | **0.003** |
|  | **hsa-miR-4443** | **-9.659** | **0.009** |
| Fibrosis 3/1 | hsa-miR-22-3p | 3.678 | 0.033 |
|  | **hsa-miR-1273f** | **-2.549** | **0.015** |
|  | **hsa-miR-1273a** | **-10.965** | **0.002** |
|  | **hsa-miR-4433b-3p** | **-11.903** | **0.001** |
| Fibrosis 4/1 | **hsa-miR-660-5p** | **4.821** | **0.010** |
|  | **hsa-miR-223-3p** | **3.772** | **0.001** |
|  | **hsa-miR-125a-5p** | **3.173** | **0.003** |
|  | **hsa-miR-16-5p** | **2.884** | **0.038** |
|  | hsa-miR-25-3p | 2.294 | 0.034 |
|  | hsa-miR-4449 | -4.143 | 0.014 |
|  | hsa-miR-760 | -5.365 | 0.049 |
|  | hsa-miR-144-5p | -6.119 | 0.021 |
|  | **hsa-miR-4443** | **-10.721** | **0.026** |
|  | hsa-miR-664b-5p | -10.913 | 0.046 |
|  | hsa-miR-2110 | -11.824 | 0.017 |
| Fibrosis 3/2 | hsa-miR-6876-5p | 6.870 | 0.043 |
|  | hsa-miR-1255b-5p | 5.674 | 0.010 |
|  | **hsa-miR-486-5p** | **-2.451** | **0.019** |
|  | hsa-miR-1246 | -2.541 | 0.020 |
| Fibrosis 4/3 | **hsa-miR-1273a** | **54.312** | **0.000** |
|  | **hsa-miR-4433b-3p** | **9.465** | **0.011** |
|  | hsa-miR-1306-5p | 4.965 | 0.046 |
|  | hsa-miR-30a-3p | 4.542 | 0.027 |
|  | **hsa-miR-660-5p** | **3.574** | **0.021** |
|  | **hsa-miR-1273f** | **2.592** | **0.029** |
|  | **hsa-miR-223-3p** | **2.587** | **0.019** |
|  | **hsa-miR-125a-5p** | **2.577** | **0.011** |
|  | hsa-miR-1260b | 2.393 | 0.032 |
|  | hsa-miR-150-3p | -2.419 | 0.043 |
|  | hsa-miR-331-3p | -7.632 | 0.027 |

**S4 Table. The fold changes of various microRNA expression according to liver fibrosis grade**

| MicroRNA |  | Fibrosis 0,1 | Fibrosis2 | Fibrosis 3 | Fibrosis 4 |
| --- | --- | --- | --- | --- | --- |
| miR-660-5p | Fold change | 1.00 | 2.51 | 1.35 | 4.82 |
|  | Standard error | NA | 0.83 | 0.87 | 0.88 |
| miR-125a-5p | Fold change | 1.00 | 1.98 | 1.23 | 3.17 |
|  | Standard error | NA | 0.50 | 0.52 | 0.56 |
| miR-122 | Fold change | 1.00 | 1.38 | -1.02 | -1.60 |
|  | Standard error | NA | 0.62 | 0.62 | 0.71 |

**Supplementary Figure legend**

S1 Fig. The expression of miR-122 is decreased in advanced liver fibrosis stages. (A) Read counts and (B) reads per million (RPM) counts by NGS according to liver fibrosis stages. (C) RPM of mir-122 patients divided into fibrosis stage 0-2, advanced fibrosis, and fibrosis stage 3-4.

S2 Fig. The expression of miR-122 is downregulated in advanced liver fibrosis stages. miR-122 expression was quantified in non-alcoholic fatty liver disease population.


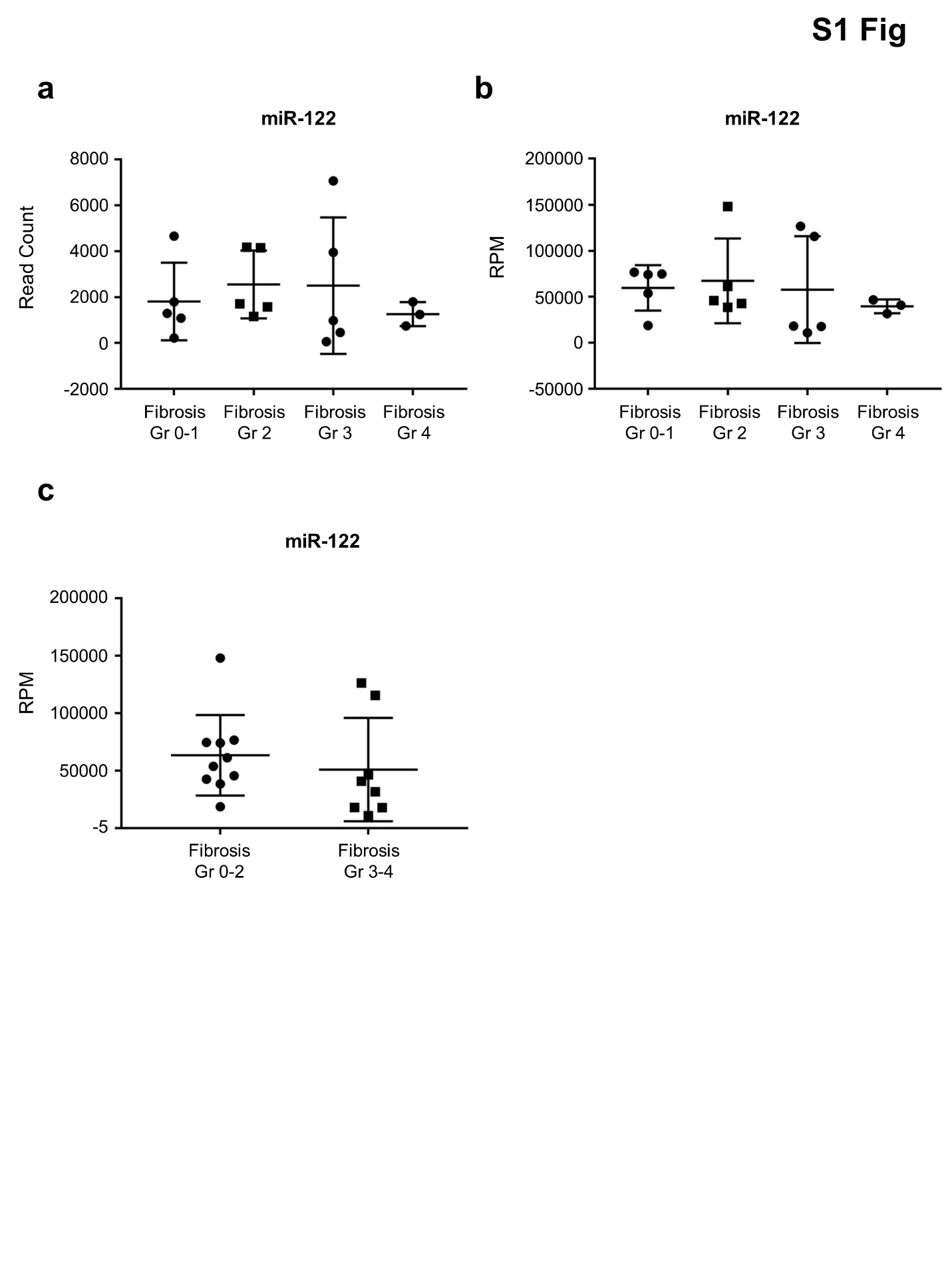


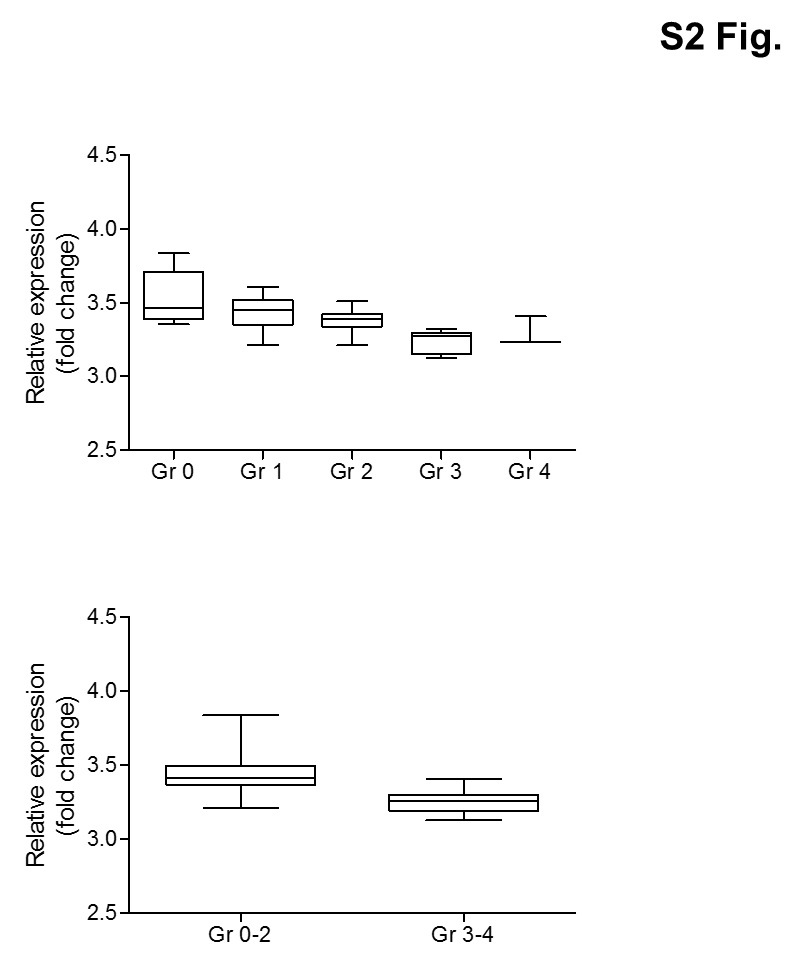


**STROBE Statement—Checklist of items that should be included in reports of *cohort studies***

|  | **Item No** | **Recommendation** | **Page No** |
| --- | --- | --- | --- |
| **Title and abstract** | 1 | (*a*) Indicate the study’s design with a commonly used term in the title or the abstract | 3 |
|  |  | (*b*) Provide in the abstract an informative and balanced summary of what was done and what was found | 3 |
| **Introduction** | | | |
| Background/rationale | 2 | Explain the scientific background and rationale for the investigation being reported | 4-5 |
| Objectives | 3 | State specific objectives, including any prespecified hypotheses | 4-5 |
| **Methods** | | | |
| Study design | 4 | Present key elements of study design early in the paper | 6 |
| Setting | 5 | Describe the setting, locations, and relevant dates, including periods of recruitment, exposure, follow-up, and data collection | 6 |
| Participants | 6 | (*a*) Give the eligibility criteria, and the sources and methods of selection of participants. Describe methods of follow-up | 6 |
|  |  | (*b*) For matched studies, give matching criteria and number of exposed and unexposed | N/A |
| Variables | 7 | Clearly define all outcomes, exposures, predictors, potential confounders, and effect modifiers. Give diagnostic criteria, if applicable | 6-11 |
| Data sources/ measurement | 8* | For each variable of interest, give sources of data and details of methods of assessment (measurement). Describe comparability of assessment methods if there is more than one group | 6-11 |
| Bias | 9 | Describe any efforts to address potential sources of bias | 6-11 |
| Study size | 10 | Explain how the study size was arrived at | 6-11 |
| Quantitative variables | 11 | Explain how quantitative variables were handled in the analyses. If applicable, describe which groupings were chosen and why | 6-11 |
| Statistical methods | 12 | (*a*) Describe all statistical methods, including those used to control for confounding | 10-11 |
|  |  | (*b*) Describe any methods used to examine subgroups and interactions | 10-11 |
|  |  | (*c*) Explain how missing data were addressed | 10-11 |
|  |  | (*d*) If applicable, explain how loss to follow-up was addressed | 10-11 |
|  |  | (*e*) Describe any sensitivity analyses | 10-11 |
| **Results** | | |  |
| Participants | 13* | (a) Report numbers of individuals at each stage of study—eg numbers potentially eligible, examined for eligibility, confirmed eligible, included in the study, completing follow-up, and analysed | 12 |
|  |  | (b) Give reasons for non-participation at each stage | 12 |
|  |  | (c) Consider use of a flow diagram | 12 |
| Descriptive data | 14* | (a) Give characteristics of study participants (eg demographic, clinical, social) and information on exposures and potential confounders | 12 |
|  |  | (b) Indicate number of participants with missing data for each variable of interest | 12 |
|  |  | (c) Summarise follow-up time (eg, average and total amount) | 12 |
| Outcome data | 15* | Report numbers of outcome events or summary measures over time | N/A |

*Give information separately for exposed and unexposed groups.

**Note:** An Explanation and Elaboration article discusses each checklist item and gives methodological background and published examples of transparent reporting. The STROBE checklist is best used in conjunction with this article (freely available on the Web sites of PLoS Medicine at http://www.plosmedicine.org/, Annals of Internal Medicine at http://www.annals.org/, and Epidemiology at http://www.epidem.com/). Information on the STROBE Initiative is available at http://www.strobe-statement.org.
