# Supplementary figures and images for: Clinical impact of serum exosomal microRNA in liver fibrosis
Source: PLoS One. 2021 Sep 10;16(9):e0255672. doi: 10.1371/journal.pone.0255672 (PMC8432846; doi:10.1371/journal.pone.0255672)

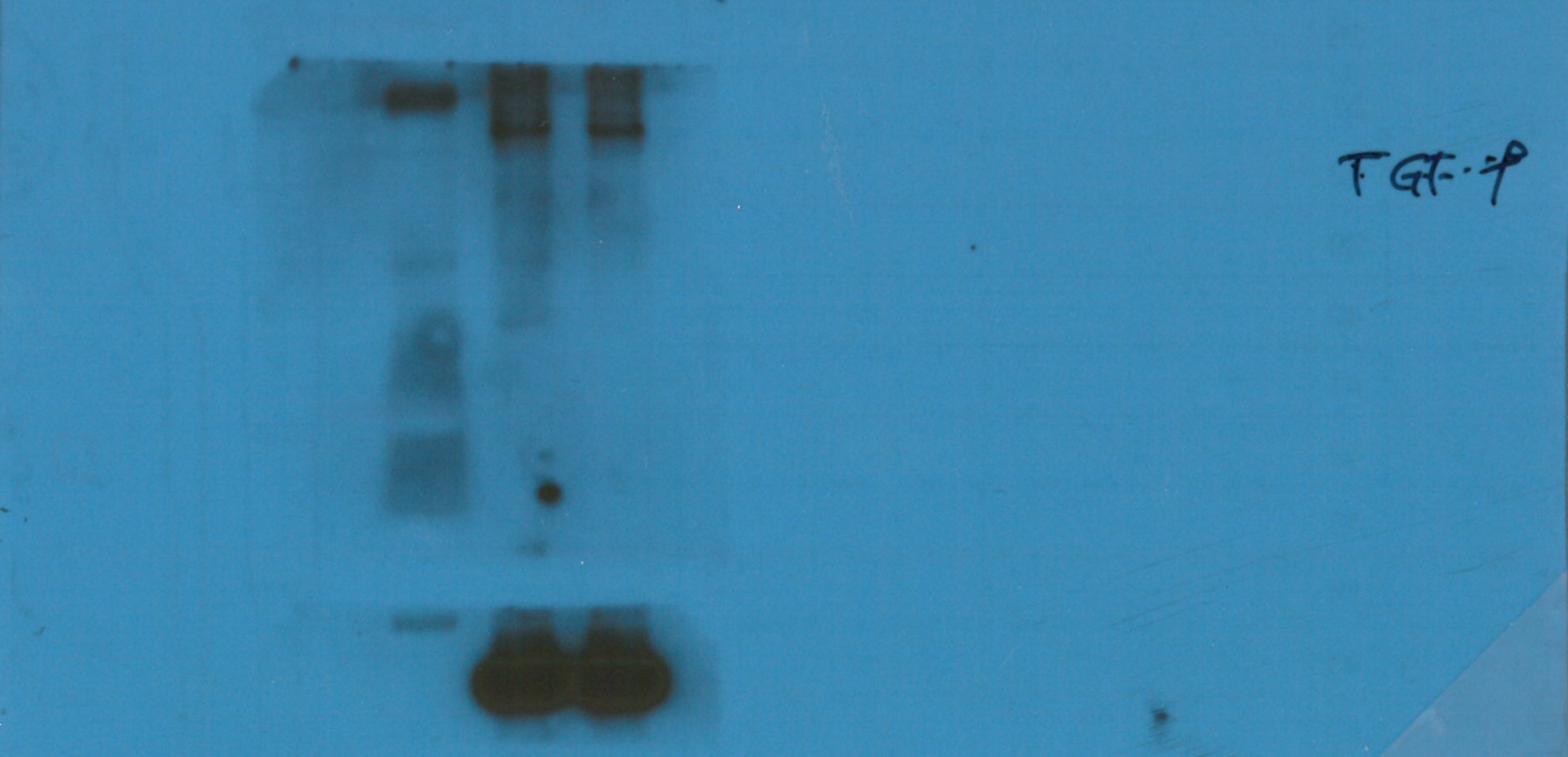

Supplement: S2 File — (ZIP) [file pone.0255672.s002.zip › TGF-beta.jpg]

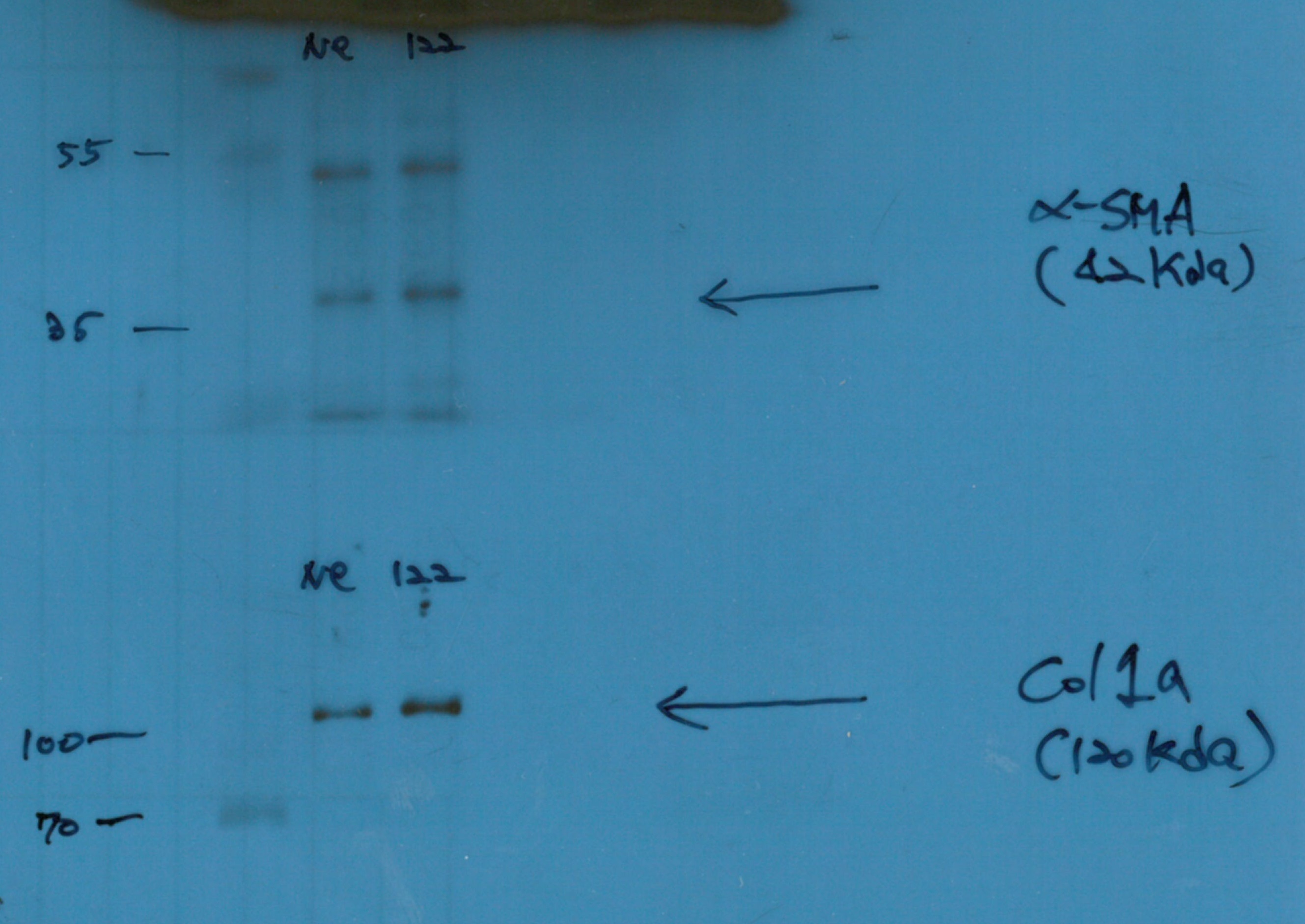

Supplement: S2 File — (ZIP) [file pone.0255672.s002.zip › A-sma_and_Col1a.jpg]

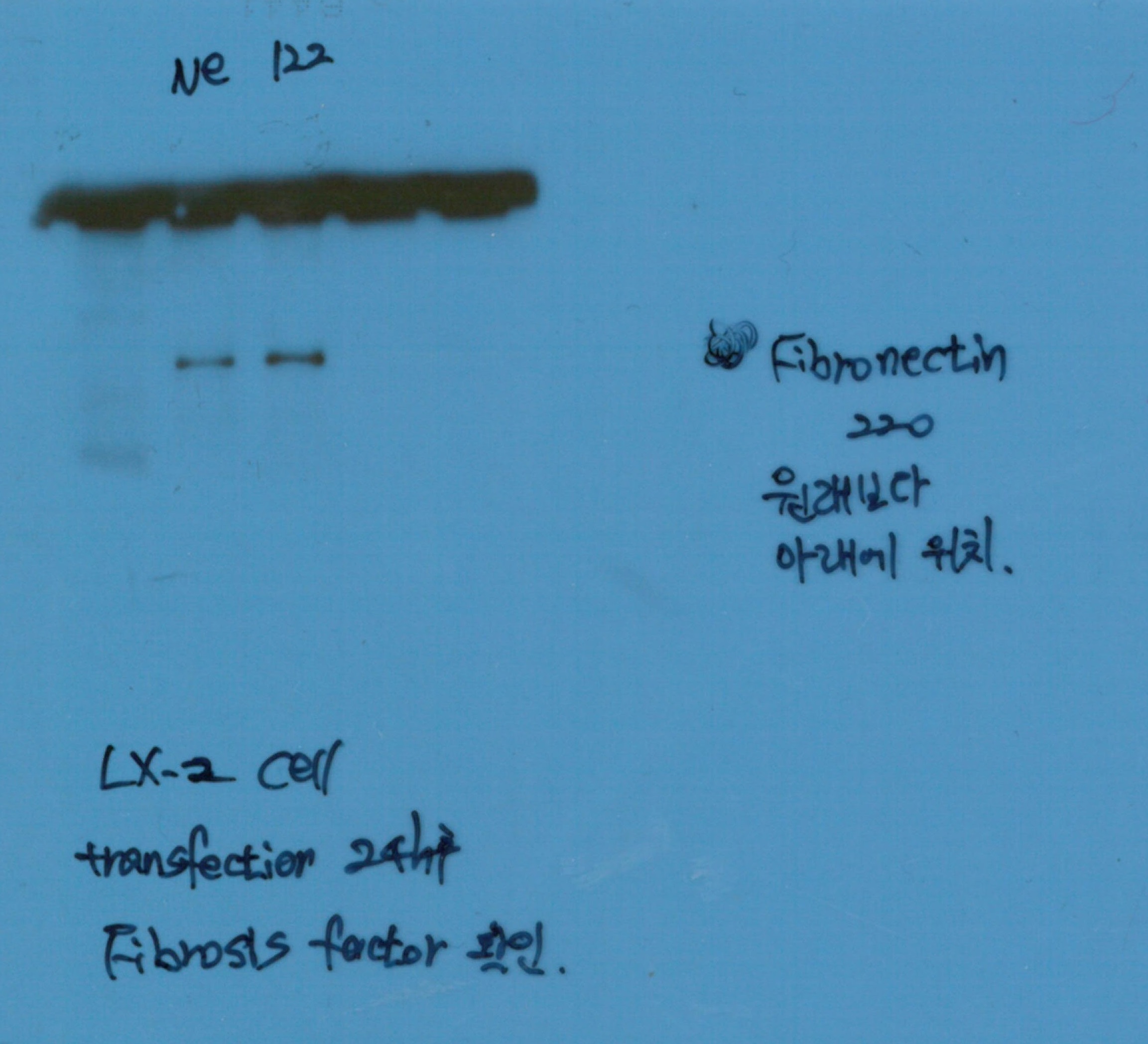

Supplement: S2 File — (ZIP) [file pone.0255672.s002.zip › Fibronectin.jpg]

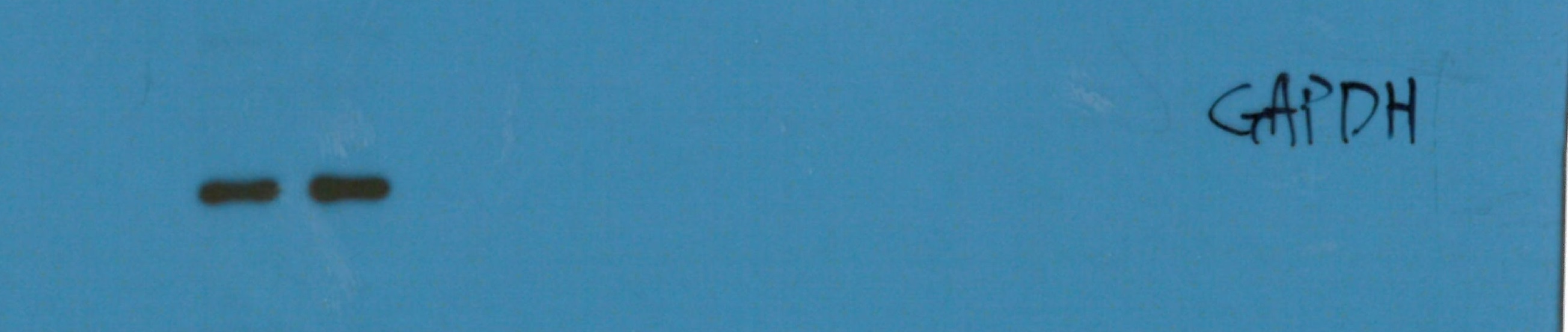

Supplement: S2 File — (ZIP) [file pone.0255672.s002.zip › GAPDH.jpg]
